# Studies on the Restriction of Murine Leukemia Viruses by Mouse APOBEC3

**DOI:** 10.1371/journal.pone.0038190

**Published:** 2012-05-29

**Authors:** Silvia Sanchez-Martinez, Amanda L. Aloia, Demetria Harvin, Jane Mirro, Robert J. Gorelick, Patric Jern, John M. Coffin, Alan Rein

**Affiliations:** 1 HIV Drug Resistance Program, National Cancer Institute, Frederick National Laboratory for Cancer Research, Frederick, Maryland, United States of America; 2 AIDS and Cancer Virus Program, SAIC Frederick, Inc., Frederick National Laboratory for Cancer Research, Frederick, Maryland, United States of America; 3 Science for Life Laboratory, Department of Medical Biochemistry and Microbiology, Uppsala University, Uppsala, Sweden; 4 Department of Molecular Biology and Microbiology, Tufts University, Boston, Massachusetts, United States of America; Institut Pasteur Korea, Republic of Korea

## Abstract

APOBEC3 proteins function to restrict the replication of retroviruses. One mechanism of this restriction is deamination of cytidines to uridines in (−) strand DNA, resulting in hypermutation of guanosines to adenosines in viral (+) strands. However, Moloney murine leukemia virus (MoMLV) is partially resistant to restriction by mouse APOBEC3 (mA3) and virtually completely resistant to mA3-induced hypermutation. In contrast, the sequences of MLV genomes that are in mouse DNA suggest that they were susceptible to mA3-induced deamination when they infected the mouse germline. We tested the possibility that sensitivity to mA3 restriction and to deamination resides in the viral *gag* gene. We generated a chimeric MLV in which the *gag* gene was from an endogenous MLV in the mouse germline, while the remainder of the viral genome was from MoMLV. This chimera was fully infectious but its response to mA3 was indistinguishable from that of MoMLV. Thus, the Gag protein does not seem to control the sensitivity of MLVs to mA3. We also found that MLVs inactivated by mA3 do not synthesize viral DNA upon infection; thus mA3 restriction of MLV occurs before or at reverse transcription. In contrast, HIV-1 restricted by mA3 and MLVs restricted by human APOBEC3G do synthesize DNA; these DNAs exhibit APOBEC3-induced hypermutation.

## Introduction

Mammals have evolved a number of “restriction” factors that function to block infection by retroviruses and other pathogens. One of these is the APOBEC3 restriction system. The best-studied member of the APOBEC3 family is human APOBEC3G (hA3G). Briefly, hA3G protein is incorporated into HIV-1 particles produced by infected cells. When these virions infect new target cells, hA3G deaminates cytidines to uridines in minus-strand DNA (the initial product of reverse transcription); this results in replacement of guanosine with adenosine in the coding strand of proviral DNA. In turn, HIV-1 encodes a protein, “Vif”, which binds to hA3G in the infected cell and brings it to the proteasome for degradation, thereby interfering with its inclusion in assembling progeny virions [1], [2]. The high frequency of G to A (“G∶A”) mutations is a major, but not the only, mechanism by which hA3G restricts HIV-1 [3], [4], [5].

Mice encode only a single APOBEC3 species (“mA3”). The overall architecture of mA3 is apparently “reversed” in mA3 relative to hA3G [6], [7]. The natural expression of mA3 is known to function to limit the spread within infected mice of both murine leukemia viruses (MLVs) [8], [9], [10], [11], which are gammaretroviruses, and mouse mammary tumor virus, a betaretrovirus [12], [13], [14]. In both of these viruses, mA3 exerts this restriction without inducing detectable G∶A mutations. Thus, it is clear that mA3 can inhibit retrovirus infection by some mechanism other than cytidine deamination; this additional mechanism is not yet understood. On the other hand, some MLV isolates are sensitive to cytidine deamination by mA3 [15], [16], [17]; it is striking that the sequences of endogenous MLV genomes, present within normal mouse DNA, do contain G∶A mutations, indicating that the MLVs that infected the mouse germline and gave rise to these endogenous virus genomes were sensitive to mA3-induced mutation when they infected the germline [18].

The effects of mA3 on Moloney MLV (MoMLV), which has a long history of passage and selection for robust replication in mice, are somewhat different from its effects on many MLVs [19], [20]. By comparing the restriction of both MoMLV and Vif-deficient HIV-1 by both mA3 and hA3G, we found that MoMLV is partially resistant to inactivation by mA3, and that inactivation of MoMLV by mA3 does not involve G∶A mutation. In contrast, mA3 induces high levels of G∶A mutation in Vif-deficient HIV-1. The mechanism of the partial resistance of MoMLV is completely unknown, but it does not involve exclusion of mA3 from the virion, or indeed from the mature core within the virion [19], [20].

As Gag is the most abundant protein in the virus particle and determines the structure of the particle, it seemed possible that some difference between the Gag proteins of MoMLV and those of endogenous MLVs might be responsible for the apparent difference in sensitivity of the viruses to mA3-induced mutation. We have tested this possibility in the present work. We created a chimeric MLV in which the *gag* gene of MoMLV was replaced by that of a polytropic endogenous MLV. This chimera is fully infectious, indicating that this “fossil” *gag* gene is fully functional. Somewhat surprisingly, the responses of this chimera to both mA3 and hA3G were qualitatively indistinguishable from those of MoMLV itself: thus Gag does not control sensitivity to restriction by these APOBEC3s. The data also show that mA3 blocks infection by MLVs before or at the initiation of reverse transcription.

## Results

### Creation of Chimeric MLV

The sequences of the “polytropic” and “modified polytropic” endogenous MLVs show clear evidence of mA3 action during infection of the mouse germline [18]. We chose PMV19, a polytropic endogenous MLV in C57BL/6 DNA, as a representative endogenous MLV; while this genome contains G∶A mutations, the Gag protein that it encodes has the consensus PMV amino-acid sequence. As described in Materials and Methods, the PMV19 *gag* gene was amplified from C57BL/6 DNA and cloned; the *gag* gene in an infectious MoMLV molecular clone was then precisely excised, from the AUG initiator codon to the UAG termination codon, and replaced with the PMV19 *gag* gene.

### Infectivity of Chimeric MLV

**Figure 1:**
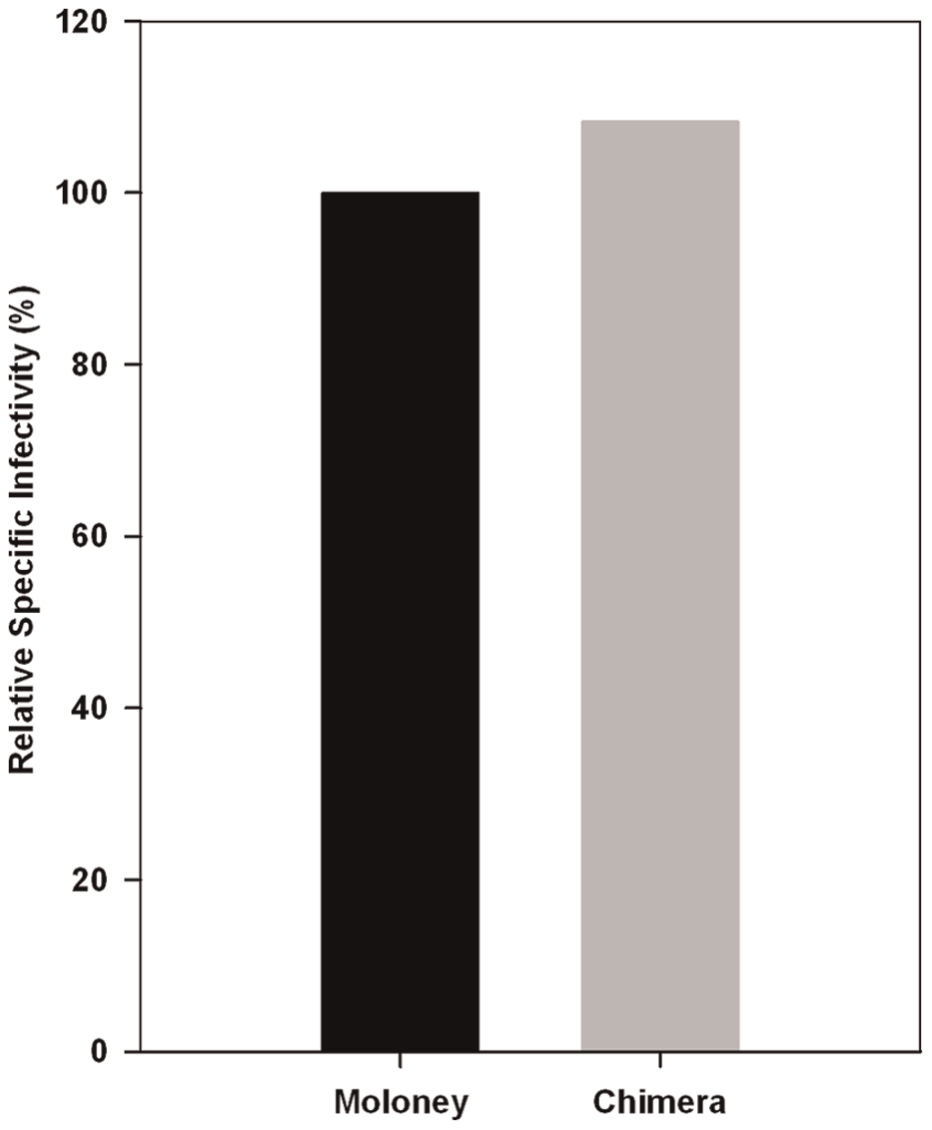
Specific infectivity of the viruses. 293T cells were transiently transfected with an infectious MoMLV or chimera proviral genome together with the reporter plasmid pBABE-Luc. 293T-mCAT1 cells were then infected with culture supernatants from the transfectants, and lysates of these cells were assayed for luciferase activity. Virions were assayed for RT activity following precipitation from the culture supernatants with polyethylene glycol. The graph shows the luciferase activity divided by the RT activity of the viruses, with the value for MoMLV set to 100%; thus the data represent the relative specific infectivities of the samples.

To test the ability of the chimeric MLV genome to produce infectious MLV particles, we transfected this molecular clone, together with pBABE-Luc, an MLV-derived retroviral vector encoding luciferase [20], into 293T cells. Our MoMLV clone was used as a control in this experiment. Culture fluids were collected from the transfected cells. The level of MLV particles in the two samples was quantitated by assaying reverse transcriptase (RT) activity; the chimera was found to produce approximately the same amount of virus as MoMLV (data not shown). Cultures of 293 cells expressing the ecotropic MLV receptor, mCAT1, were then infected with these samples and assayed for luciferase activity. As shown in Fig. 1, the specific infectivity of the chimeric virus, as measured by the ratio of luciferase activity to RT activity, was virtually identical to that of the MoMLV control.

### Restriction of Chimeric MLV by mA3 and hA3G

**Figure 2:**
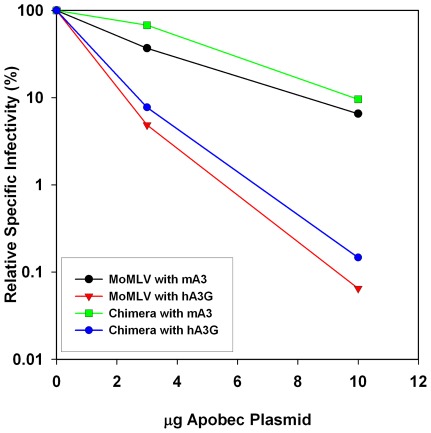
Effects of the different APOBEC3s on the infectivity of MoMLV and chimera viruses. Virus particles were produced by transient transfection of viral clones together with pBABE-Luc and 0, 3, or 10 μg of either mA3 or hA3G expression plasmids. Specific infectivities were calculated as described in Materials and Methods, dividing the luciferase activity values by the RT activity values; specific infectivity of samples produced without APOBEC3 is set to 100%. Black line, MoMLV with mA3; green, chimera with mA3; red, MoMLV with hA3G; blue, chimera with hA3G. Results are plotted *vs.* the quantity of APOBEC3 plasmid used in the transfections to generate the viruses.

To test the susceptibility of the chimeric MLV to restriction by mA3 and hA3G, we co-transfected the chimeric MLV clone with pBABE-Luc and different doses of plasmids encoding the two APOBEC3s; again, the MoMLV clone was tested in parallel. As shown in Fig. 2, the chimeric MLV is inactivated by mA3, but appears to be far more sensitive to hA3G than to mA3; the curves showing the inactivation of the chimeric virus by both mA3 and hA3G are very similar to those for MoMLV itself.

**Figure 3:**
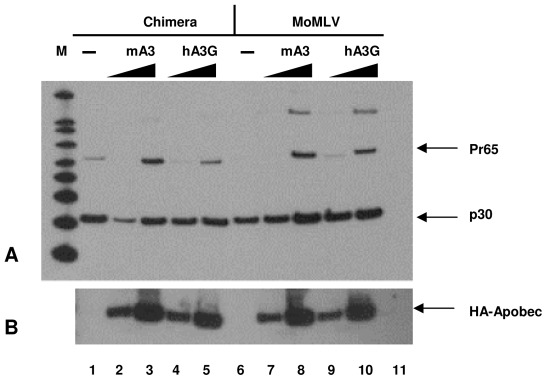
Immunoblotting of virus particles. A) Western blot on produced virus. Chimera (lanes 1 to 5) or MoMLV (lanes 6 to 10) were prepared by transient transfection of 293T cells, using 0, 3, or 10 μg APOBEC3 DNA as well as viral DNA; control cells were transfected with DNA of pGCcos3Neo (a derivative of pSV2Neo) (lane 11). The culture supernatants were fractionated as described in Materials and Methods and equal volumes of culture fluid were loaded into the lanes and analyzed by immunoblotting against P30^CA^. M, molecular weight markers. **B) Encapsidation of different APOBEC3s in MoMLV and chimera viruses.** Virus particles shown in figure 3A were analyzed by immunoblotting against the HA tag. Equal volumes of culture fluid were loaded into the lanes.

We also tested the incorporation of mA3 and hA3G proteins into chimeric MLV particles by immunoblotting. Samples of chimeric MLV and MoMLV, prepared by transfection together with 0, 3, or 10 μg of mA3 or hA3G plasmid, were analyzed; as both mA3 and hA3G are tagged with a hemagglutinin (HA) epitope, their levels can be compared in a single immunoblot using anti-HA antiserum. Profiles of the virion preparations, analyzed with a broadly reactive anti-CA antiserum, show that there were similar levels of virus in all of the samples (Fig. 3A). As shown in Fig. 3B, the two APOBEC3 proteins are packaged at similar levels in the two viruses; virus made in the presence of 10 μg of APOBEC3 plasmid contains more APOBEC3 protein than that made in 3 μg plasmid. Fig. 3A also shows that the APOBEC3 proteins interfere to some extent with the normal processing of Pr65^Gag^ during virus maturation.

### Effect of APOBEC3s upon Viral DNA Synthesis

The point in the MLV replication cycle that is blocked by mA3 is not known. We tested the ability of MoMLV and the chimeric MLV, produced in the presence of mA3 or hA3G, to synthesize viral DNA upon infecting new cells. The viruses used in these assays were produced by transfection of 293T cells that had previously been stably transfected with pLXSH, an MLV-derived vector encoding the hygromycin phosphotransferase (*hph*) gene. This vector is rescued into infectious particles by the viral proteins encoded by the MLV plasmids. The released virions were then used to infect new 293-mCAT cells and assayed for their ability to synthesize *hph* DNA. This protocol helps to eliminate the background representing DNA from plasmids used to produce the viruses.

**Figure 4:**
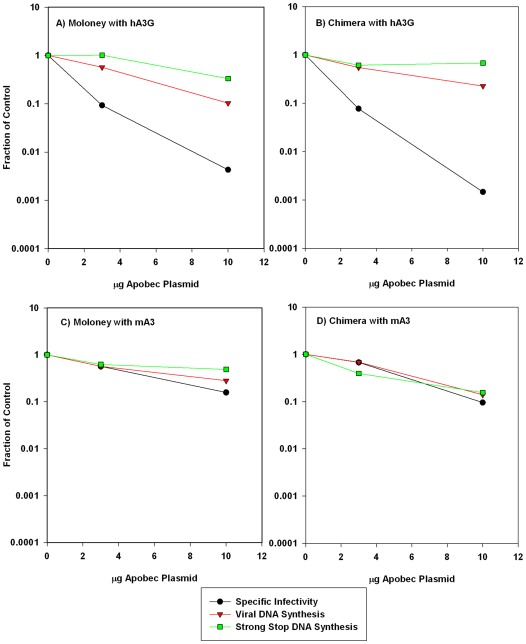
Comparison of the infectivity of the viruses with the synthesis of the viral DNA. Viruses were prepared by co-transfection of 293T-hygro cells (which contain pLXSH) with viral clones, pBABE-Luc, and 0, 3, or 10 μg of APOBEC3 DNA. 293-mCAT1 cells were then infected with the resulting culture fluids. Relative specific infectivities (black lines) were determined as in Fig. 2. Parallel cultures were lysed and assayed for *hph* DNA (red lines) and strong stop DNA (green lines) as described in Materials and Methods. Values in each assay were divided by the value for the virus produced with no APOBEC3 plasmid.

Viruses were produced by transient co-transfections of the pLXSH-bearing cells with plasmids containing viral genomes; the MLV-derived luciferase vector pBABE-Luc; and APOBEC3s. The viral populations produced by the transfected cells will include some particles with luciferase-vector genomes and some with pLXSH genomes (as well as some with MLV genomes). Infectivities were quantitated by the luciferase assay, as in Fig. 2 above, and the infected cells were lysed and assayed for *hph* DNA by real-time PCR. Results of these assays are shown in Fig. 4. With hA3G (Fig. 4A and 4B), the loss of infectivity in both MLVs was far greater than the reduction in viral DNA synthesis. In contrast, mA3 inhibited viral DNA synthesis by both viruses to virtually the same extent as it inhibited infectivity (Fig. 4C and 4D). Somewhat similar results with MoMLV were presented earlier [20].

We also assayed the cell lysates for minus-strand strong-stop DNA, the initial product of reverse transcription (Fig. 4, green lines). In all cases, the reduction in strong-stop DNA closely resembled the reduction in total DNA, as assessed by the *hph* values.

**Figure 5:**
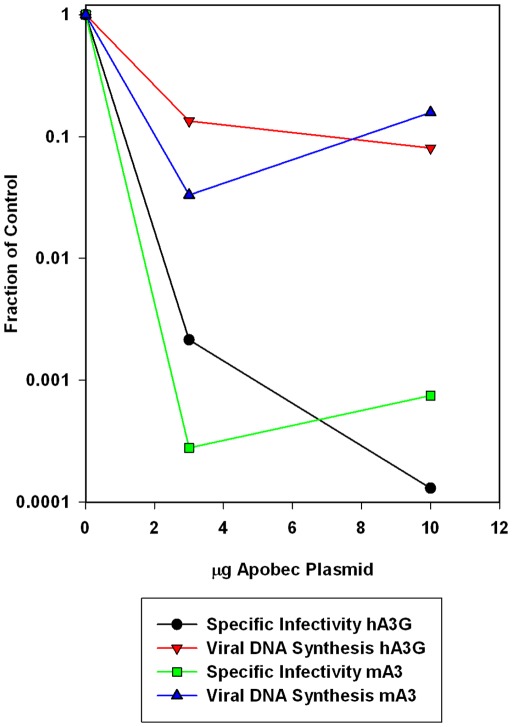
Effect of APOBEC3s on ΔVif HIV-1 DNA synthesis. 293-mCAT1 cells were infected with ΔVif HIV-1. Infectivity and RT activity were assayed as described [25], [29], [30]. Twenty-four hours after infection, the cells were lysed and assayed by real-time PCR for *Luciferase* DNA (black and green lines) as described in Materials and Methods [32]. Specific infectivity is represented with red and blue lines.

As a control, we also tested the effect of the APOBEC3s on DNA synthesis by, as well as infectivity of, ΔVif HIV-1. As shown in Fig. 5, the two APOBEC3s were very similar in their effects on ΔVif HIV-1: in both cases, the reduction in infectivity far exceeded the reduction in DNA synthesis.

### Sequence Analysis of Viral DNAs Synthesized in Presence of APOBEC3s

The results in Fig. 4 show that both MoMLV and the chimeric MLV have not entirely lost the ability to undergo reverse transcription when they infect fresh cells, despite the presence of mA3 or hA3G in the virions. We also performed sequence analysis of these DNAs to look for G∶A mutations, which would be evidence of cytidine deamination in minus-strand DNA. A stretch of *hph* DNA was amplified from the cell lysates and PCR products were individually cloned and subjected to sequence analysis. As shown in Table 1, the chimera, like MoMLV [20], does not undergo a notable increase in the frequency of G∶A mutations when it is inactivated by mA3. In contrast, hA3G increases this frequency >30-fold. The high level of G∶A mutations seen in the assays of DNA produced by MLV particles containing hA3G (Table 1, 5^th^ to 7^th^ and 11^th^ and 12^th^ rows) shows that our experimental techniques are suitable for detection of these mutations.

**Table 1:** G to A mutation frequencies.

Virus	APOBEC3 Plasmid (μg)	Relative Specific Infectivity	Nucleotides Scored	G:A Mutations	Mutation Frequency	Increase in Frequency
**Moloney**	None	1	27,086	7	0.00026	1
	1 mA3	0.6578	20,018	10	0.0005	1.9
	3 mA3	0.4327	19,712	9	0.00046	1.8
	10 mA3	0.1571	24,825	30	0.0012	4.6
	1 hA3G	0.1381	29,954	217	0.0072	27.9
	3 hA3G	0.04814	24,106	299	0.012	47.7
	10 hA3G	0.00131	29,698	450	0.015	57.7
**Chimera**	None	1	12,783	9	0.00070	1
	3 mA3	0.6762	9,792	7	0.00071	1.02
	10 mA3	0.09567	12,160	3	0.00025	0.35
	3 hA3G	0.07715	8,832	198	0.022	32.0
	10 hA3G	0.00146	11,532	277	0.024	34.3

## Discussion

It has been previously reported that MoMLV, a laboratory strain of MLV previously subjected to extensive selection for replication in mice, is partially resistant to restriction by mA3 and fully resistant to G∶A mutation induced by mA3 [19], [20]. The mechanism by which mA3 restricts MoMLV without cytidine deamination is unknown, as is the mechanism by which MoMLV evades mA3 restriction: its resistance, unlike the mechanisms by which other retroviruses resist APOBEC3s, does not involve exclusion of mA3 from the assembling virion.

The sequences of “polytropic” and “modified polytropic” endogenous MLVs show clear evidence of cytidine deamination by mA3 at the time that they were inserted into the mouse germline [18]. It seemed possible that the resistance of MoMLV to mA3 is attributable to its Gag protein, and that the sensitivity of endogenous MLVs could be traced to a difference between their Gag proteins and that of MoMLV. However, we found (Fig. 2) that a chimeric MLV, identical to MoMLV except that its *gag* gene was from a polytropic endogenous virus, showed the same responses to mA3 (and hA3G) as MoMLV. In future experiments, we will determine whether sensitivity to mA3 can be mapped to the *pol* gene of a sensitive MLV. Many MLVs also produce an alternative, N-terminally extended and glycosylated form of the Gag protein, called “glyco-Gag” [21], [22]; we will also test whether, as recently suggested [23], this protein is involved in mA3 resistance.

As the mechanism by which mA3 inactivates MLVs is not known, it was of interest to determine whether these viruses can undergo reverse transcription when they infect new host cells. We found that the extent to which MoMLV and the chimeric MLV lost infectivity under the influence of mA3 was very similar to the extent to which the viruses lost the ability to synthesize viral DNA (Fig. 4). Moreover, MLVs inactivated by mA3 are evidently unable to synthesize even minus strand strong-stop DNA, the initial product of reverse transcription (Fig. 4, green lines). Thus, mA3 interferes with infection by MLVs either before or at the initial stages of viral DNA synthesis. In contrast, restriction of MLVs by hA3G and of ΔVif HIV-1 by mA3 is not at the level of DNA synthesis, as the degree of viral inactivation in these systems is far greater than the inhibition of DNA synthesis (Fig. 4A and 4B, Fig. 5). The viral DNAs synthesized in these cases are, however, characterized by very high levels of G∶A mutation; thus cytidine deamination is presumably a major contributor to virus inactivation in these cases. Taken together, these results highlight the distinctive nature of restriction of MLVs by mA3.

## Materials and Methods

### Construction of chimeric MLV

To generate a chimeric MLV, we replaced the *gag* gene in our infectious clone of MoMLV [20] with the *gag* gene of the endogenous MLV PMV19 [18]. Using the specific primer flank-7 (5′-GGCAGGAGCCAGGTGTAATGG-3′) that anneals in the PMV19 flanking region and a reverse primer PMV19R1 (5′-GGGGGGCTCCTGACCCTGACCTCCCTAGTCACC-3′), that anneals at the end of PMV19 *gag*, the PMV19 *gag* sequence was isolated from C57BL/6 mouse DNA (Jackson Laboratory, Bar Harbor, ME).

The chimeric DNA was created using the sequential PCR [24] approach. The specific PMV19 *gag* sequence and the MoMLV proviral genome were used as templates; the amplification primers contain 5′ extensions that are homologous to a portion of the other target gene. Specifically, the sequences of these primers were 5′-**TTT CTG TAT TTG TCT GAG AAT** ATG GGA CAG ACC GTA ACT-3′ (PMV19F2); 5′- **GAA TTC TGA AAG ACC CCA CCT GTA GG**-3′ (MoMLV F1); 5′-AGT TAC GGT CTG TCC **CAT ATT CTC AGA CAA ATA CAG AAA**-3′ (MoMLV R1); 5′- CTA ACC TTA GGT GAC TAG **GGA GGT CAG GGT CAG** -3′ (MoMLV F2); and 5′-**GTC GAC AAA GAG TTC AAA GGG**-3′ (MoMLV R2). MoMLV-derived sequences are shown in bold lettering here. Details of cloning will be provided upon request.

### Cells and viruses

Virus particles were produced by transient transfection of 293T cells, or 293T cells that had previously been stably transfected with pLXSH plasmid DNA, as previously described [20], [25], [26]. Plasmids expressing either hA3G or mA3 were a kind gift from Nathaniel Landau (New York University School of Medicine). The proteins were both tagged at their C termini with the hemagglutinin (HA) epitope [27]. The mA3 protein encoded by the plasmid used here is the isoform lacking exon 5. HIV-1 was prepared as previously described [20], [25].

### Luciferase activity assay

293 cells expressing mouse cationic amino acid transporter 1 (mCAT1, the receptor for ecotropic MLVs; a kind gift of J. Cunningham, Harvard Medical School) [28] were infected with the filtered culture supernatants. Forty-eight hours after infection, cell extracts were assayed for luciferase activity with the Luciferase Assay System (Promega, Madison, WI) as previously described [20], [25].

Luciferase assays were performed in triplicate; the three values were, in general, within 10% of each other.

### Reverse Transcriptase (RT) Assay

The samples were analyzed for RT activity as previously described [29], [30] following concentration with polyethylene glycol (PEG) [30]. RT assays were performed in triplicate and the three values were, in general, within 10% of each other. “Specific infectivity” is the mean of the luciferase values divided by the mean of the RT values.

### Immunoblotting

Virus particles were isolated from filtered culture fluids by centrifugation through 20% sucrose (w/w) in phosphate-buffered saline at 110,000×*g* for 1 hour at 4°C. The virus pellet was resuspended in 2xNuPAGE sample buffer (Invitrogen, Carlsbad, CA).

Immunoblotting against MLV p30^CA^ was performed with rabbit polyclonal anti-MLV p30^CA^ antiserum and against HA-tagged APOBEC3 proteins with mouse anti-HA monoclonal antibody 16B12 (Covance, Princeton, New Jersey) as previously described [20]. Western Lightning Plus-ECL (Perkin Elmer, Waltham, MA) was used for detection.

### Analysis of viral DNA synthesis

The ability of MLV-derived virus particles to perform DNA synthesis upon infecting new host cells in the presence or absence of APOBEC3 proteins was assayed as previously described [20], [31], [32], [33].

Viruses were produced by transient transfection of 293T hygro cells [26] as mentioned above. The culture supernatants were treated after filtration with 10 U/ml of DNAse 1 (Ambion, Austin, TX) and 4 mM MgCl_2_ for 1 h at 37°C to eliminate contaminating plasmid DNA from the virus before infecting 293-mCAT1 cells. An aliquot of the DNAse-treated virus was inactivated by incubation at 68°C for 20 min and used as control in the infection. Twenty-four hours after infection, the cells were lysed and the genomic DNA was extracted by the QIAamp DNA Mini Kit (Qiagen, Hilden, Germany). The genomic DNA was then treated with DpnI (New England Biolabs, Ipswich, MA) for 1 h prior to PCR amplification to further eliminate contaminating parental plasmid DNA. DNA copy numbers obtained with the heated virus were indistinguishable from those obtained by “infecting” 293-mCAT1 cells with culture fluid from mock-transfected 293T hygro cells, and were <10^−4^ of the values obtained in the infected cultures.

AmpliTaq Gold polymerase and associated buffer were purchased from Applied Biosystems/Life Technologies (Foster City, CA). A master mix of PCR reagents was prepared such that the final concentrations in the PCRs for *hph* DNA were: 1xPCR buffer; 300 nM dNTPs; 2.5 mM MgCl_2_; 500nM HPH680F; 500 nM HPH742R; 100 nM 701 probe [31] and 0.06 U/μl polymerase. Final concentrations of PCR reagents for *Luciferase (luc)* DNA were: 1xPCR buffer; 300 nM dNTPs; 2.5 mM MgCl_2_; 1000 nM LucF; 1000 nM LucR; 260 nM Luc probe [32] and 0.06 U/μl polymerase. Reactions were heated to 95°C for 10 minutes followed by 40 cycles of 95°C for 15 seconds and 60°C for 60 seconds. Final concentrations of PCR reagents for Strong stop DNA were; 1xPCR buffer; 200 nM dNTPs; 4 mM MgCl_2_; 600 nM MLV-SSF4 (5′-CGTGTATCCAATAAACCCTCTTGC-3′); 600 nM MLV-SSR2 (5′-GCTGACGGGTAGTCAATCACTC-3′); 50 nM P-SSMLV-1 probe (5′Fam-ATCCGACTTGTGGTCTCGCTGTTCCT-Tamra 3′) and 0.06 U/μl polymerase. Reactions were heated to 95°C for 10 minutes followed by 40 cycles of 95°C for 30 seconds and 60°C for 30 seconds.

All reactions were performed using a DNA engine Opticon 2 instrument (MJ Instruments, now BioRad, Hercules, CA). DNA copy numbers were measured in triplicate and the three values were, in general, within 20% of each other. In the plots of “DNA synthesis”, the mean of the copy numbers is divided by the mean of the RT values.

### G to A hypermutation

DNA was collected from 293-mCAT1 cells that had been infected with virus carrying the pLXSH vector as described above. The *hph* DNA was amplified from 100 ng of the genomic DNA with hph 2050F (5-AAAGCCTGAACTCACCGCGACGTC-3) and hph 3030R (5-CACGAGTGCTGGGGCGTCGGTTTC-3) primers by using T*aq* polymerase (Invitrogen, Carlsbad, CA) for 35 cycles under the following PCR conditions: 95°C, 45 s; 67.5°C, 1 min; and 72°C, 1 min. The PCR products were cleaned up using G-50 columns (GE Healthcare, Buckinghamshire, United Kingdom). The 1-kb PCR product was then ligated into TOPOTA PCR 2.1-TOPO (Invitrogen, Carlsbad, CA) and transformed into Top 10 cells following the manufacturer's conditions. Colonies were selected by growth on ampicillin-containing medium. In order to minimize repeated amplification and cloning of the same DNA, transformed bacteria were only grown for 30 min before plating. Selected colonies were grown in 1 ml Terrific Broth as described before [20]. The purified DNAs were sequenced with the M13R primer.

Sequence data were analyzed for mutations by trimming all sequences to the same length using *MEGA* version 4 [34] and aligning them with ClustalW (EMBL-EBI at http://www.ebi.ac.uk/clustalw/#).
